# Pigmented contact cheilitis: a systematic review

**DOI:** 10.4317/medoral.26484

**Published:** 2024-10-13

**Authors:** Amanda dos Santos Figueiredo, Gabriel Lima Braz, Francielli Fernandez Garcia, Laura Barreto Moreno, Alini Cardoso Soares, Camila Barcellos Calderipe, Ana Carolina Uchoa Vasconcelos

**Affiliations:** 1 Diagnostic Center of Oral Diseases, Dental School, Federal University of Pelotas - UFPel, Pelotas, Rio Grande do Sul, Brazil; 2 Department of Oral Diagnosis, Piracicaba School of Dentistry, Campinas State University- UNICAMP, Piracicaba, São Paulo, Brazil

## Abstract

**Background:**

Our objective was to carry out a systematic review of available data regarding pigmented contact cheilitis (PCC).

**Material and Methods:**

Electronic searches were performed using PubMed, Scopus, Embase, Web of Science and LILACS electronic databases. The risk of bias was assessed using the Joanna Briggs Institute tool.

**Results:**

A total of 2070 articles were retrieved, with 7 of them reporting PCC cases. Female individuals (*n*=6/85.7%) were more affected, with a mean age of 32±15.4 years (range: 22-47 years). Ricinoleic acid and gum ester were the most frequently observed allergic compounds, each present in two cases. Three patients had lesions in both lips (42.8%), while three other patients (42.8%) had lesions only in the lower lip. All reported cases presented with multiple pigmented lesions (*n*=7/100.0%). Associated symptoms involved itching, scaling, swelling, erythema, vesicles and papules. The mean evolution time was 13.5±15.6 months (range: 2-36 months), and the average follow-up time was 12±0 months.

**Conclusions:**

This is a condition that often scares the patient due to the unexpected appearance of hyperpigmentation. For this reason, the information transmitted in this review is expected to be relevant so that the health professional can include PCC in their list of differential diagnoses.

** Key words:**Cheilitis, hyperpigmentation, contact dermatitis.

## Introduction

Pigmented contact cheilitis (PCC) occurs when there is frequent and repeated contact with a small amount of a sensitizer. Inflammatory manifestations are usually not evident, and hyperpigmentation occurs secondarily. Basal liquefaction degeneration and pigmentary incontinence are responsible for this melanin pigmentation (1-3). The most common sensitizers are found in lipstick ingredients, although they can also be found in other products that come into contact with the lips, including foods and oral care products (3-5).

It is hypothesized that saliva contributes to the dilution or elimination of allergens resulting in lip involvement without intraoral findings (2). The primary clinical feature of PCC is multiple brown macules that may involve both lips as well as surrounding skin (3). Other associated symptoms include itchiness, swelling, and scaling (5,6). The diagnosis of PCC is confirmed after performing a patch test, which is the gold standard for detecting allergenic substances (7,8). After confirming the contact reaction, the patient should be instructed to avoid using any products containing the detected allergenic substance (9). In some cases, topical corticosteroids may be used to alleviate other associated symptoms (10,11).

PCC raises significant concern for the patient and represents a diagnostic challenge for dental surgeons. Considering the importance of summarizing the knowledge of this challenging diagnostic condition, we aimed to conduct a systematic review of the available clinicopathological data on PCC to answer the question: What are the demographic, clinical, and histopathological characteristics of PCC?

## Material and Methods

- Eligibility criteria

Inclusion criteria were based on the PECOS (Population, Exposure, Comparison, Outcomes, Studies Design) acronym as follows: P) Patients with PCC; E) Cosmetics (including lipsticks, lip gloss, and salves/balm), personal hygiene products (such mouthwashes and toothpastes), and food (including chewing gums and candies); C) Not applicable; O) clinical, demographic and histopathological characteristics of patients with a diagnosis of PCC; S) Observational and intervention studies.

Exclusion criteria were as follows: 1) Studies that did not prove the presence of PCC when using the patch test; 2) Reviews, book chapters, letters, personal or expert opinions, and meeting abstracts; 3) Studies for which full texts were not available;

- Information sources and search strategies

Electronic searches without restrictions of publication date were performed in June 2024 in the following databases: PubMed/MEDLINE (National Library of Medicine), Scopus (Elsevier), Embase (Elsevier), Web of Science (Clarivate Analytics), and LILACS (Virtual Health Library). The Gray literature was also searched in Google Scholar and ProQuest. Personalized search strategies were performed for each bibliographic database (Supplement 1). Additionally, a manual search of bibliographies and reference lists of selected studies was performed to identify any publications that may have been missed in the electronic searches. The references found were imported to the EndNote software (Clarivate Analytics, Philadelphia, USA), where duplicates were removed after identification.

- Selection process

The titles and abstracts of all articles found through the searches were read by two authors independently (A.S.F., and G.L.B.). The calibration of the authors was analyzed by assessing the agreement among the two reviewers regarding the evaluation of titles and abstracts of the first 50 references encountered in the searches. A 0.90 Kappa value demonstrated excellent agreement between reviewers. The two authors assessed the references after calibration. If the title and abstract met the inclusion criteria, the article was selected for a comprehensive reading of the full text. Full texts of articles with titles and abstracts lacking information for a definitive decision were acquired. After evaluation of the full texts, articles that satisfied the eligibility criteria were selected. Divergent opinions regarding inclusion or exclusion between A.S.F. and G.L.B. were resolved by discussions with a senior lecturer in Oral Medicine (A.C.U.V.).

- Data extraction

The following data were extracted from each article included when available: authors’ name, year and country of publication; study design; individual’s age and sex; allergic diseases; PCC allergenic compounds; PCC clinical features [anatomical location (lower lip/upper lip/both) and number of lesions (single/multiple)]; PCC-associated signs and symptoms; time of evolution (months); follow-up (months); histopathological features (yes/no).

- Study risk of bias assessment

The Joanna Briggs Institute - University of Adelaide tool for case reports was used to evaluate the included articles (12). The case reports selected were evaluated according to the following parameters: Patient’s demographic characteristics clearly described; Patient’s history clearly described and presented as a timeline; Current clinical condition clearly described; Diagnostic tests or assessment methods and the results clearly described; The intervention(s) or treatment procedure(s) clearly described; The post- intervention clinical condition clearly described; Adverse (harmful effects) or unanticipated events identified and described; The case report provides takeaway lessons. The parameters were answered as “yes” (low risk of bias), or “no” (high risk of bias).

- Data synthesis 

Statistical analysis was performed using the Statistical Package for the Social Sciences (SPSS) for Windows, version 25.0 (IBM Corporation, Armonk, NY). Due to a lack of methodological uniformity in the included studies, a meta-analysis of the results obtained was not feasible. Therefore, the results were instead descriptively summarized in this review.

## Results

**Figure 1:**
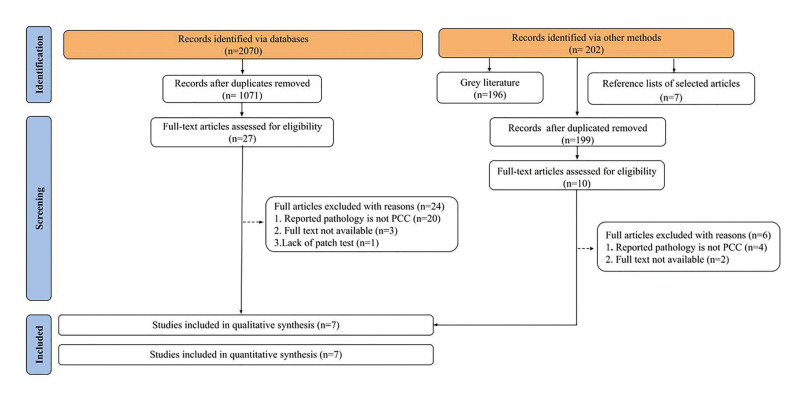
Flow diagram of a literature search adapted from PRISMA (2020).

The searches identified 2070 articles in the five databases and 202 additional records by other methods, for a total of 2272 studies. After eliminating duplicates, 1270 references remained, 1071 from the main databases and 199 obtained by other methods. Screening by titles and abstracts resulted in 37 articles selected for reading in full which were subsequently excluded for the following reasons: reported pathology was not PCC (*n*=24), full text not available (*n*=5), and lack of patch test (*n*=1) (Supplement 2). A total of 7 case reports met the selection criteria and were included in the present review. The flowchart describes the search and selection process (Fig. 1).

- Study characteristics

Demographic and clinical data are presented in Table 1 (Supplement 3). All cases included occurred in Asia (*n*=7/100.0%) across four countries. Mean age at diagnosis was 32±15.4 years (range: 22-47 years). Six (85.7%) patients were females and one (14.3%) was a male. Regarding allergic diseases, systemic lupus erythematosus and allergic rhinitis were related in one case (14.2%) each. Among the allergenic compounds identified as causing PCC, ricinoleic acid was present in two cases (25.0%) and ester gum was also responsible for two cases (25.0%). Other compounds found included dipentaerythritol fatty acid ester (*n*=1/12.5%), nickel (*n*=1/12.5%), paraphenylene diamine (*n*=1/12.5%), and propylgallate (*n*=1/12.5%). The anatomical location of the PCC in three patients was in both the lower and upper lips (*n*=3/42.8%), another three other patients (*n*=3/42.8%) had lesions only in the lower lip. In one patient (14.2%) the lip location was not reported. Other signs and symptoms of PCC found were: desquamation (*n*=4/26.6%), itching (*n*=3/20.0%), swelling (*n*=3/20.0%), erythema (*n*=2/13.4%), papules (*n*=2/13.4%), and vesicles (*n*=1/6.6%). Mean evolution time was 13.5±15.6 months (range: 2-36) and mean follow-up time was 12±0 months. Six (85.7%) cases did not present a histological aspect while only one (14.3%) described the histological aspect of the lesion. A complete description of the PCC case is provided in Supplement 4.

- Risk of bias in studies

**Figure 2:**
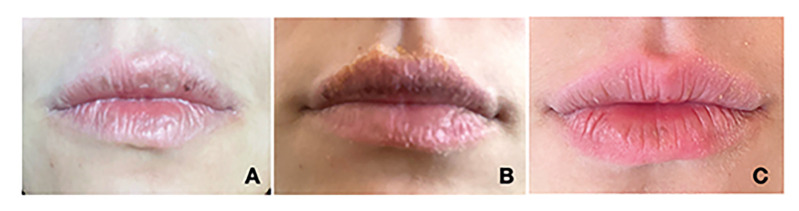
Pigmented Contact Cheilitis (PCC) caused by carmine in a 23-year-old female. The patient reported a four-month history of multiple gray-to-smoky hyperpigmentation on the lips, with initial symptoms including lip peeling, itching, and dryness (A/B). Subsequently, there was an improvement in the condition noted five months after discontinuing the use of the dye (C).

Critical appraisal of the case reports revealed that all articles had a clear description of the patient's demographic characteristics. In most cases, a clear description of the patient's history and timeline was not provided. However, most reports clearly described the patient's current clinical condition. All articles demonstrated clarity in the description of diagnostic tests, evaluation methods and results obtained. Treatment interventions or procedures were clearly described in most cases. In most cases, the intervention condition was not informed and adverse events did not occur. Additionally, all articles provided takeaway lessons (Supplement 5, Fig. 2).

## Discussion

Cheilitis” is a term derived from Greek word "cheilos" (χεῖλος), which means “lips”, and the Latin suffix “-itis”, commonly used to indicate inflammation. This term denotes a non-specific inflammation of the lips, necessitating further specific descriptors for an accurate diagnostic definition (13). Although several types of cheilitis are described, most of the literature does not provide clear recommendations for classification (10). Pigmented contact dermatitis (PCD) was first described in 1969 by Osmundsen, who observed pigmentation resulting from contact dermatitis to an optical brightener (14). Some authors describe PCC as a variant of PCD, with the distinction that PCD occurs on the skin, while PCC specifically affects the lips (6,15,16). In any case, both are still considered to be a variation of contact dermatitis (CD), which involves sensitizing components and inflammatory characteristics, but no pigmentation (1). In this context, it's important to recognize that atopic dermatitis (AD), a multifactorial pruritic eczematous condition, shares similar clinical findings and can occur concomitantly with CD (17-20).

The pigmentary condition in CD was initially described in Asia (14,21), where reports continue to emerge, with ethnicity and lack of product legislation appearing to be linked (22,23). As observed in the present study, PCD and allergic cheilitis usually occurs in women between the third and fifth decades of life (4,22,24-26). These demographic findings align with a population more exposed to lip cosmetics (24). A previous history of dermatitis, allergic rhinitis and other contact allergies, which are also prevalent among young women, may involve some susceptibility to the development of PCC (27,28). Patients affected by these conditions have a reduced inflammatory threshold, which makes the skin more prone to inflammatory and pigmentary reactions (28-30).

Although the etiopathogenesis of PCC/PCD has not been completely understood, there is some evidence that repeated exposure to low levels of certain substances can cause a delayed (type IV) hypersensitivity reaction (22). Additionally, the role of AD in PCC etiology is not clear (30,31). Kang *et al*. (2018) conducted a retrospective review to explore the association between labial pigmentation and AD. Interestingly, the authors observed that AD patients with labial pigmentation had significantly more frequent allergic disorders and had higher IgE levels than AD patients without labial pigmentation, suggesting the role of other immune dysregulation beyond delayed hypersensitivity in the development of these lip macules. However, it is important to note that while the authors excluded any local or systemic factors related to labial pigmentation, it is difficult to determine whether they represent a true PCC in the absence of a patch test. In the present survey, allergic/immunologic disease were detected in only two cases (28.6%), and none of them reported AD. Unfortunately, the low number of cases in the present study make it difficult to draw precise and reliable comparisons.

Personal care products and cosmetics are the most common cause of PCC due to the many potentially sensitizing substances in their composition (2,3,8). In this review, most cases were associated with substances present in lipsticks and lip balms, including ricinoleic acid, ester gum, dipentaerythritol fatty acid ester, and propyl gallate (16,32,33). Previous studies using patch testing on patients with allergic contact cheilitis also identified patients' own lipsticks as the primary source of contact allergens (34-36). Although the profile of causative allergens has varied among the studies and appears to have shifted over time; metals, oils, and fragrances are frequently related to positive reactions in allergic cheilitis (24,25,28,30). Interestly, in the present survey, one case was related to green tea with the associated substance being nickel (5). Nickel is a common sensitizer that can be found in dental devices, instrument mouthpieces, piercing jewelry, makeup containers, food, drinks, and cosmetic products (37). More than 4000 allergens have been associated with hypersensitivity reactions (38,39). This data could explain the lack of a standardized consensus regarding patch tests to investigate allergic cheilitis (24), making it difficult or impossible to estimate which or how many substances are associated with PCC.

Pigmentation occurs in the area exposed to the compound; in the case of lipstick, pigmentation usually occurs on both lips (6,16,32). On the other hand, if the substance has sufficient contact with only one lip, pigmentation will occur solely on that lip, often resulting in multiple pigmentations (5,11). Diffuse brownish lip pigmentation can be found in several adult-onset conditions that could mimic PCC, including those with and without systemic involvement, such as Addison’s disease and Laugier-Hunziker syndrome, respectively. The presence of abnormal systemic symptoms and signs such as weakness, anorexia, and weight loss, as well pigmentation in other anatomical locations besides the lips, can help differentiate these conditions (3). Moreover, the distribution, duration of pigmented lesions, as well as drug treatments, and changes in the pattern are paramount for the differential diagnosis of numerous pigmented lesions that affect the lips (30). For all cases in this review, the clinical diagnosis was based on the presence of hyperpigmentation of the lips and confirmation of the allergenic substance using a patch test.

The gold standard for the diagnosis of PCC is the patch test (7). The technique is performed using a series of allergens distributed in small amounts in patches that are attached to the patient's skin, most commonly on the back (38,40). Although not pathognomonic, histopathological findings of PCC are important to exclude other diagnostic hypotheses. Liquefaction degeneration of the basal layer and pigmentary incontinence are the main histopathological features associated with PCD (1,41). Histological pigmentary incontinence results in a natural tattoo of the melanin pigment, which is absorbed so slowly that the hyperpigmentation caused by this process becomes persistent (1). In the present sample, a biopsy was performed in only one case, revealing the increased melanophages in the upper dermis as the main histopathological characteristic (5).

The evolution time of PCC seems to vary according to the exposure to these sensitizers (41). Curiously, the shortest evolution time observed in the present systematic review was 4 months. Due to the sudden appearance of patches on the lips, patients usually seek for evaluation as soon as possible. Our data align with the findings of Kanokrungsee and colleagues (2023), who observed that 77 (58.8%) of 131 individuals with allergic contact cheilitis had an evolution time presented more than three months (24). While discontinuing the use of products that cause the contact reaction usually resolves common symptoms, pigmentation can persist for longer periods, as observed in the present review (22). Thus, a follow-up period may be required, especially in cases where the patch test could not be performed.

The present systematic review has some limitations that should be recognized. First, there is a lack of standardization of the PCC nomenclature. Second, information about follow-up was not available in most cases. The main reason for that limitation was the lack of protocols used to describe patient details among the studies reviewed. Therefore, it is strongly recommended that case reports use tools such as CARE guidelines (for CAse REports) and Strengthening the Reporting of Observational studies in Epidemiology (STROBE) to provide more standardized information. Third, the lack of clinical studies other than case reports is a noTable concern. Last, the small sample precluded a more powerful statistical analysis, such as meta-analysis.

Considering the findings of the present review, PCC mainly affects women in the third decade of life and is caused by the use of lip cosmetics. Clinically, it presents as multiple macules associated with desquamation, itching, and swelling. Furthermore, our limited histological findings reinforce that a detailed medical history and a thorough physical examination are fundamental, since the diagnosis of PCC is primarily clinical, and biopsies are not necessary. This condition often alarms the patient due to the unexpected appearance of hyperpigmentation. Therefore, the information provided in the present report is expected to be relevant for health professionals to include PCC in their list of differential diagnoses.

- Protocol and registration

This systematic review was conducted according to the guidelines of the Preferred Reporting Items for Systematic Reviews and MetaAnalyses (PRISMA) Statement (42). A protocol was drafted and registered in the International Prospective Register of Systematic Reviews (PROSPERO). The following number was assigned to the present systematic review: CRD42023420111.

## Figures and Tables

**Table 1:** Compiled demographic and clinical characteristics.

Variable **		n (%)
Continent (n=7)	Asia	7 (100.0)
Age in years (n=7)	Mean (SD)	32(±15.4)
	Range	22-47
Decades of life	20-29	3 (42.8)
	30-39	2 (28.6)
	40-49	2 (28.6)
Sex (n=7)	Female	6 (85.8)
	Male	1 (14.2)
Allergenic/Immunological Diseases (n=7)	Absence of allergic/immunological diseases	3 (42.8)
	Not related	2 (28.6)
	Systemic lupus erythematosus	1 (14.2)
	Allergic Rhinitis	1 (14.2)
PCC Allergenic Compounds (n=8)*	Ricinoleic acid	2 (25.0)
	Ester gum	2 (25.0)
	Dipentaerytritol fatty acid ester	1 (12.5)
	Nickel	1 (12.5)
	Paraphenylene diamine	1 (12.5)
	Propyl gallate	1 (12.5)
PCC Clinical Features (n=7): Anatomical location (n=7)	Lower and upper lip	3 (42.8)
	Lower lip	3 (42.8)
	Unreported lip	1 (14.2)
PCC Clinical Features (n=7): Number of lesions (n=7)	Multiple	7 (100.0)
PCC associated signs and symptoms (n=15)*	Desquamation	4 (26.6)
	Itching	3 (20.0)
	Swelling	3 (20.0)
	Erythema	2 (13.4)
	Papules	2 (13.4)
	Vesicles	1 (6.6)
Time of evolution, in months (n=3)	Mean (SD)	13.5(±15.6)
	Range	2-36
Follow up, in months (n=3)	Mean (SD)	12(±0)
Histopathological features (n=7)	No	6 (85.7)
	Yes	1 (14.3)

** Numbers represent the reported data for each variable; * These variables were not counted by the number of cases, but rather by the number of times they were mentioned in each case report; SD: standard deviation.
